# Stimulation Parameters of Manual Acupuncture and Their Measurement

**DOI:** 10.1155/2019/1725936

**Published:** 2019-08-28

**Authors:** Ruoyun Lyu, Ming Gao, Huayuan Yang, Zonglin Wen, Wenchao Tang

**Affiliations:** School of Acupuncture-Moxibustion and Tuina, Shanghai University of Traditional Chinese Medicine, Shanghai 201203, China

## Abstract

The therapeutic effect of manual acupuncture (MA) is closely related to the stimulation amount. In the clinical studies, the stimulation amount is often difficult to be determined. The reason is that there are many parameters affecting the stimulation amount, including manipulation selection, treatment time, needling velocity, and force, and no complete and reasonable scheme is available for the measurement of stimulation parameters. This paper reviewed the theoretical and laboratory measurement studies on MA stimulation, summarized 4 types of available parameters according to the theory of physics, and compared the advantages and disadvantages of the existing methods of parameter measurement. Such efforts are hoped for providing reference for the establishment of the stimulation parameter system of MA and possible technical solutions for future measurement experiments.

## 1. Introduction

As an ancient treatment method, the clinical effect of manual acupuncture (MA) has been proved in different ways, including randomized control trials [1, 2] and basic experiments [3, 4]. According to the theory of acupuncture, the stimulation of MA comes from specific finger manipulation that drives the translation, rotation, or tremor of the needle [5]. Therefore, it has been reported that different MA manipulations can produce different therapeutic benefits [6–10]. For example, a comparative study of lifting-thrusting and twirling manipulations showed that both types of skills can decrease the blood pressure and heart rate of the healthy volunteers, but the needling sensation caused by lifting-thrusting is stronger with better intervention effect [11]. Furthermore, even with the same manipulation, the physiological changes or physicochemical reactions of organisms are diverse because of different stimulation parameters such as frequency and depth. Several studies have suggested the twirling method with variable frequencies had the respective effects on gastric motility amplitude [12] and gastric vagal afferent fiber discharge [13]. The different needling depth was also compared for the relief of muscle pain, and the result showed that electrical pain thresholds of the muscle group (depth of 10 mm) increased higher than those of the skin group (a depth of 3 mm) [14]. In clinical studies, the effect of deviation of same acupuncture prescription exists extensively because the different stimulation parameters were selected by different acupuncturists. This phenomenon had also been proved in some quantitative studies of MA manipulation [15–17]. Based on this situation, it is difficult to determine whether the subjects of current clinical or experimental research of MA strictly accept the stimulation amount prescribed by the experiment protocol.

Compared with MA, the electroacupuncture is easier to control due to its four specific stimulation parameters, including waveform, frequency, time, and current intensity [18], and its stimulation amount can be adjusted accurately [19]. Based on these characteristics, electroacupuncture is being adopted in more and more clinical treatments [20, 21] and scientific research studies [22, 23] of acupuncture. Therefore, summarizing and measuring stimulation parameters of MA is an active area of research, as it is critical for the determination of stimulation amount of MA and its clinical application and promotion.

According to the operation method of MA, it can be attributed to a kind of physical stimulation driven by finger movements [24, 25]. Some parameters for human motion measurement such as kinematic parameters [26] (including amplitude, velocity, acceleration, angle, angular velocity, angular acceleration, and frequency) and kinetic parameters (force) [27] can provide reference for the establishment of the parameter system of MA. This paper will review the current progress of quantitative research on MA manipulations, analyze and classify the available parameters, and look forward to the future trends of corresponding measurement technologies.

## 2. Theoretical Research of Stimulation Parameters

Throughout history, the stimulation parameters of MA have been mentioned in some ancient medical books. For example, the force of MA can be controlled by the velocity of needling with proportional relation [28]. Depth and velocity were often discussed for their effect of stimulation intensity, in brief, faster and deeper manipulation generates stronger stimulation [28, 29]. Some books even divided the MA manipulations into several types such as “mild reinforcing-attenuating,” “reinforcing,” and “attenuating” based on the velocity and amplitude of needling [29, 30].

However, lack of parameter classification and quantitative analysis is the biggest issue in ancient medical books. Contemporary studies paid more attentions on the establishment of parameter system according to the theory of physics. The “Acupuncture Manipulation Metrology” proposed by Shi [31] has defined four major stimulation elements (parameters) of MA, including force direction, force magnitude, manipulation time, and time interval between treatments, some recommended combinations of treatment elements were also provided for clinical practice. Due to the different movement characteristics, some scholars regarded that the main stimulation parameters for the lifting-thrusting method and twirling method are “insertion depth, frequency, and duration” [32] and “rotation angle, frequency, and duration” [33, 34, respectively. In terms of the force parameters, Fangjie et al. [35] proposed 3 types of force generated by the fingers operating the needle handle (lifting-thrusting force, twirling force, and swing force), which control the needle to make up-and-down, rotational, and swing motion accordingly. Moreover, in actual operation, the needle handle is often subjected to a combined effect of two or three forces. The paper of Langevin et al. [36] considered the “de qi” reaction has a biomechanical component “needle grasp,” and it is a measurable biomechanical phenomenon associated with manipulation of MA. Except needle grasp, the friction was also identified as an important parameter related to de qi during interaction between needle body and tissues [37].

Although the rigorous parameter system for MA has not been established, some studies or books have applied some parameters to guide education and clinical treatment. The university textbook *Techniques of Acupuncture and Moxibustion* [38] of China has specified the parameters and the corresponding reference range. For example, the amplitude and frequency of the “lifting-thrusting” method should be about 3∼5 mm and about 60 times per minute, respectively, while the torsion angle of needle during the “twirling” method should be between 180° and 360° once. Yuan [39] further graded the stimulation amount of MA with two parameters “amplitude and frequency,” the small stimulation amount should be <5 mm in amplitude and <90 times/min in frequency; the large stimulation amount should be >10 mm in amplitude and >120 times/min in frequency. Nevertheless, the rigorous data support and related information of measurement experiments of above parameter combinations have not been found yet.

## 3. The Measurement of Stimulation Parameters

According to the theory of physics [40], the stimulation parameters of MA can be divided into four types, “manipulation parameter,” “kinematic parameter,” “kinetic parameter,” and “time parameter.” The manipulation parameter is the acupuncture manipulations selected by acupuncturist according to the patient's diagnosis, for example, the lifting-thrusting or twirling method. The measurement of time parameters is relatively easy and can be completed by various types of timing devices, so this paper will detail research progress on measurement methods of kinematic and kinetic parameters.

### 3.1. The Measurement Research of Kinematic Parameters

**Figure 1:**
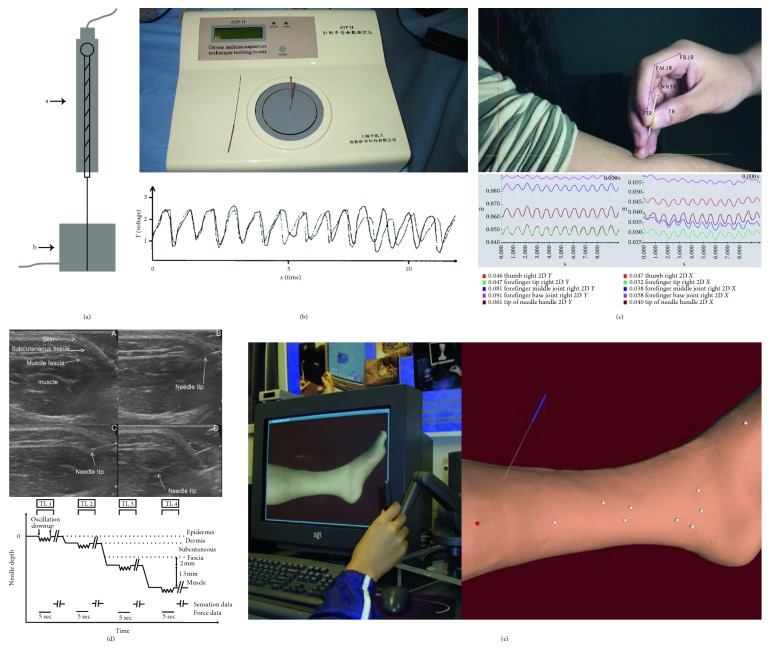
Different measurement methods for kinematic parameters of MA. (a) The modification illustration of needle body, motion sensors can be placed at needle handle (a) or needle body (b). (b) Model ATP-I acupuncture manipulation parameter determination apparatus, its voltage-time curve generated by MA manipulation shows the parameters “cycle” and “frequency.” (c) Two-dimensional motion analysis of the needle and the joints of operation fingers carried out by Tang et al.'s different curves show the kinematic parameters of different markers, respectively. (d) The motion of the needle in the tissue taken by ultrasound imaging in the study of Park et al., which can clearly determine the position of the needle tip for the measurement of depth and display the corresponding curve. (e) The tactile acupuncture manipulation simulation system can output the amplitude and velocity of needle, as well as the components along the needle.

Kinematic parameters include speed, acceleration, amplitude, and angle. A common solution for measurement is to modify needle body by adding a multiaxis motion sensor. Leow et al. [41] attached a 3-axis electromagnetic motion sensor to the top of the needle handle and obtained the kinematic parameters such as displacement and angle during the twirling method. The motion sensor was also placed at the acupuncture point in contact with needle body by Seo et al. [42, 43] for acquiring amplitude data in real time. In addition to the use of existing sensors, there are secondary developments based on existing technologies for measurement too. The “AcuSensor technology” was invented by Davis et al. [16], including a motion sensor around the tube for needle insertion, and two main motion components, “displacement” and “rotation,” were detected. Guo et al. [44] designed a sensor based on the digital signal processor (DSP) chip; it was integrated into the needle body for obtaining angle and displacement data. The modification illustration of needle body is shown in Figure 1(a).

The application of sensor technology realized the real-time acquisition of the relevant kinematic parameters of MA, but since the adhesion of sensor increased the weight and volume of needle, which affected the finger sensation of acupuncturist, some researchers chose to modify the operating environment around needle to obtain kinematic parameters without affecting operation. The “acupuncture manipulation tester,” developed by Gu [45], collected the lifting-thrusting and twirling signals with a micromotor sensor around the needle. The wave data derived from the two-channel physiological recorder can reflect the velocity change during needling. Yang et al. [46, 47] developed the “model ATP-I acupuncture manipulation parameter determination apparatus” using variable resistance sensor technology to measure the line displacement, angular displacement, and frequency of the needle (Figure 1(b)).

Although the modification of operating environment solved the issue of finger sensation, MA was performed on the machine, which still has some differences from the operation on human body, for instance, the deficiency of “de qi” sensation. In recent years, with the continuous development of motion-tracking technology based on visible or infrared light, a large number of kinematic parameters can be collected in real time without interference. An optoelectric camera was adopted by Li et al. [48] for capturing the real-time displacement and time course associated with each lifting and thrusting. Tang et al. [15] carried out a two-dimensional motion analysis of the needle body as well as the joints of operational thumb and index finger based on the motion videos of MA (Figure 1(c)). In addition to obtaining the parameters such as amplitude, velocity, and acceleration of the needle and joints, the angle and angular velocity of joint were also obtained. Optical motion capture technology was used by Zhang et al. [49] to obtain the parameters of needle and fingers, and the measured results had also been applied to the data mining, training, and assessment of MA manipulations. The joint angles of fingers were also measured by Yang et al. [50] with similar technology in both the lifting-thrusting and twirling method. Their results showed that the lifting-thrusting was mainly performed by the dorsiflexion of the wrist joint with the angle 7.23° ± 1.87°, while the twirling manipulation was mainly performed by the flexion of the interphalangeal joint of the index finger, and the angle of the 1st and 2nd interphalangeal joints was 28.33° ± 2.18°, 10.43° ± 1.69°. Apart from optical tracking, the needle body movement can also be tracked by ultrasound imaging, and the amplitude and frequency can be obtained during needle insertion and manipulation at both superficial and deep acupuncture points. The anatomical structures around the needle are seen clearly in Figure 1(d) [51–54].

Moreover, virtual technology is also used for the quantitative analysis of the needle motion. Due to the complete digital characteristic of this technology, the parameter measurement is relatively simple. For example, the tactile acupuncture manipulation simulation system developed by Leung et al. [55, 56] based on computer virtual imaging technology was used for students to practice MA manipulations (Figure 1(e)). A three-dimensional virtual environment can be established by this system with realistic haptic feedback and real-time parameter display. Similar studies were also reported by Liu et al. [57, 58] and Heng et al. [59]. However, because fingers operate on the virtual needle, the sensation is quite different from the actual operation.

### 3.2. The Measurement Research of Kinetic Parameters

**Figure 2:**
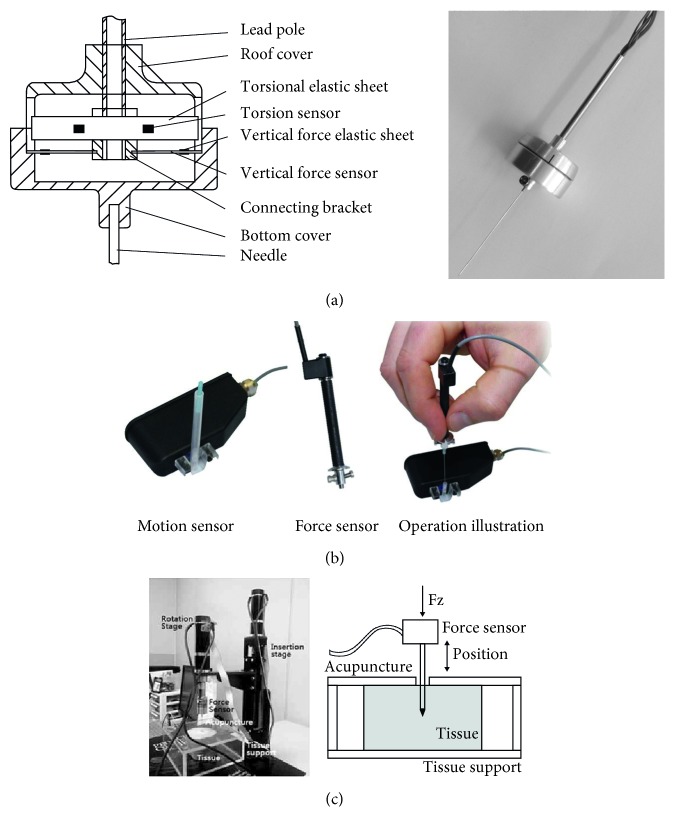
Measurement for kinetics parameters of MA based on force sensor. (a) The micromechanical sensor-attached measuring needle developed by Li et al. and corresponding modification illustration of the needle handle. (b) Motion and force sensor used in the study of Davis et al. (c) Measurement environment and schematic diagram in the study of Son et al.

The kinetic parameters of MA are usually the forces along different axes and the corresponding power. Because the existence of force is inseparable from the interaction of objects, the measurement of kinetic parameters was mainly based on various types of mechanical sensors. The micromechanical sensor-attached measuring needle of acupuncture developed by Li et al. [60] can record the frequency, velocity, and components along vertical and coronal axis during the lifting-thrusting and twirling methods (Figure 2(a)). Micromechanical sensing technology was selected by many other studies too [16, 33, 48, 61] (Figure 2(b)). According to the movement characteristics of basic manipulation, the different micromechanical sensors mainly detect the component along three axes during lifting-thrusting, as well as the torsion and tangential force of twirling. Son et al. [37] further calculated the friction coefficient of tissue to the needle body with a modified Karnopp friction model based on the measured value during the lifting-thrusting method (Figure 2(c)). The dynamic detection system developed by Ding [62] was used for measuring the interaction force between the acupuncturist and patient on needle during each kind of manipulations. Researchers also found that the waveforms and values of force and torsion could further identify the different manipulations reversely [63, 64]. Due to the attachment of micromechanical sensors, the needle body must be modified; therefore, most of the kinetic measurement studies have impacts on the finger sensation during needling inordinately, and the experimental technologies needs further improvement. Simultaneously, there has been no report on the analysis of the power generated by different force and relevant comparative study.

## 4. Future Work

The main target of future research work is to establish a stimulation parameter system and carry out related research with this system. The approach for this target is the verification of the influence of each parameter on the therapeutic effect with more reasonable measurement technology. The big data accumulation of MA based on this system can provide a quantitative reference for the analysis of the effect study such as “de qi,” and the characteristic summary of different MA manipulations. At present, the corresponding research work has also begun, for instance, the identification of the “de qi” reaction based on the friction coefficient or grip strength of the needle body [36, 37] and the characteristic comparison of MA manipulations “reinforcing” and “attenuating” with kinematic parameters such as velocity and time course [15, 42, 48].

According to the above-mentioned parameter types, some existing but still innovative technologies can provide solution options for the future work. For the measurement of kinematic parameter, markerless motion capture can be taken into consideration. Through the motion capture with high-speed camera, not only the movement of the needle body but also the motion of finger joints of acupuncturist can be quantitative analyzed in three-dimensional space [65, 66]. More importantly, it does not require the placement of any markers on the human's body, which results in maintaining the naturalness of the tester's movement [67, 68]. Therefore, this technology has been widely used in the analysis of various sports projects [69] and is very suitable for finger movement research. For example, a markerless finger position capture device “Leap Motion Controller” has been mature and start selling. Several studies about the finger tracking [66], hand gesture recognition [70, 71], and robot-arm design [72, 73] were based on it. Some more precise and professional markerless motion-tracking systems are also used to investigate the movement of fingers or handheld implements [74, 75]. Because the essence of MA is movement of needle and finger joints [76], the markerless motion capture technology can not only measure the MA on the human body but also eliminate the external interference of acupuncturist, which is the most consistent with the actual clinical situation and recommended for the measurement of kinematic parameters.

In terms of the measurement of kinetics parameter, because the mechanical measurement needs to be based on sensor, the modification of the needle body becomes inevitable. Compared with the size of the needle, the microsensing devices used in the existing researches are still too large, and the modified needles are significantly different from the actual clinical operation needles. Therefore, the needle body needs to be attached with smaller and thinner sensors. Nanomechanical sensors can be one option of the solutions and have been used for imaging force fields [77], sensing forces with tiny particles [78, 79], etc. They can be attached to the surface of the needle body to obtain kinetic parameters without significantly changing the shape, sensation, and quality of the needle body. Another alternative approach is surface electromyography (SEMG) of the forearm. SEMG refers to the collective surface electric signal from muscles, which is generated during muscle contraction and commanded by the nervous system [80]. The quantitative relationships between SEMG and muscular force have been proved early [81], and the amplitude of the SEMG should increase proportionately with the square root of the tension [82]. Therefore, the data of SEMG can also be regarded as a kinetic parameter and represent muscular force of fingers during needling. The advantage of the forearm SEMG is that the measuring electrode is located on the forearm and does not interfere with finger movements. There have been many reports on the measurement of the extensor and flexor muscles of the fingers using SEMG [83, 84], and mature measurement solution for finger motion has also been provided [85].

## 5. Conclusion

**Figure 3:**
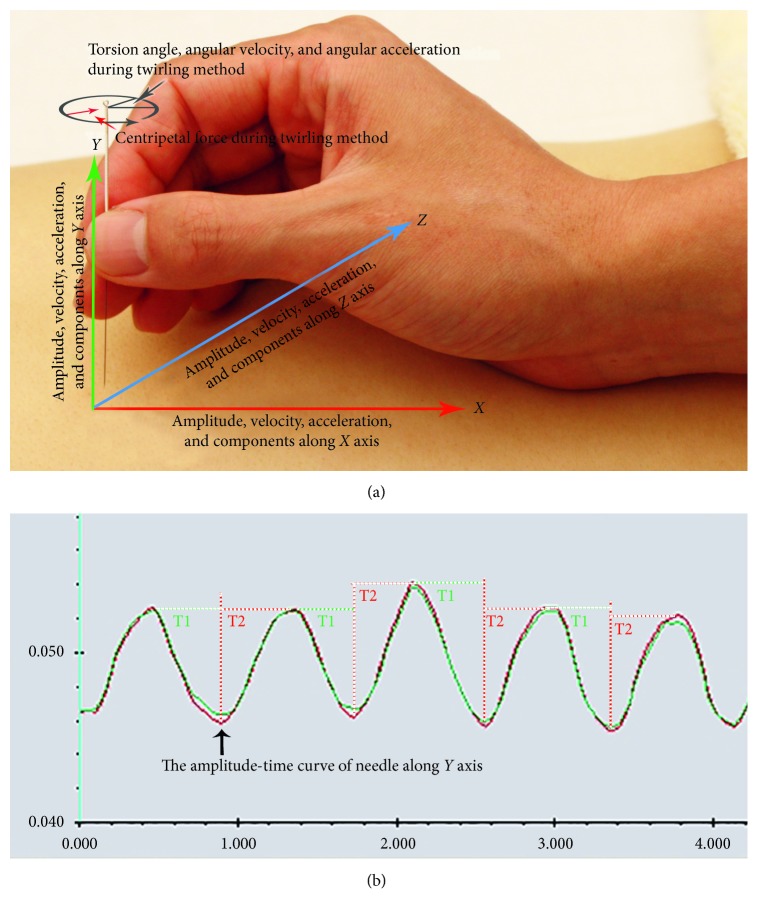
(a) Amplitude, velocity, and acceleration along *X*, *Y*, and *Z* axes. Moreover, the torsion angle, angular velocity, angular acceleration, and centripetal force can also be derived during the twirling method. (b) Amplitude-time curve of needle along the *Y* axis using motion-tracking technology, T1 is the time course of the thrusting or left twirling and T2 is the time course of the lifting or right twirling during “lifting-thrusting” or “twirling” method, respectively. Manipulation cycle is the sum of T1 and T2, and manipulation frequency is the reciprocal of cycle.

According to the current research progress, we summarize four types of parameters that can be the reference for the establishment of the stimulation parameter system of MA, as shown in Table 1, and the meaning of some parameters in this table has be illustrated in Figure 3.

In terms of the parameter measurement, the contemporary quantitative analysis of stimulation parameters of MA shows the characteristics of multiangle and interdisciplinarity, which has achieved certain breakthroughs, but it still exposes many problems. First, although sensor technology can obtain relevant kinematic and kinetic parameters in real time, the modification of the needle body for sensor attachment affects the operational sensation of fingers. Second, the measurement experiment based on the sensors surrounding the needle was performed on an apparatus instead of the human body, which is inconsistent with the actual clinical work. Third, the motion-tracking technology can complete the quantitative analysis of the stimulation parameters of MA without modifying the needle and the surrounding environment, but only kinematic parameters can be acquired, and it is easy to generate measurement errors. At the same time, the micromarkers attached on the fingers may also slightly interfere with the operation. Finally, the resistance encountered by the needle inside human tissue during operation is still hard to be obtained without invasion and interference. The comprehensive comparison of different measurement technologies has been shown in Table 2. From the above, the current bottleneck lacks a complete solution for the measurement of kinematic and kinetics parameters of MA without affecting operation and modifying environment. However, markerless motion capture, nanomechanical sensor, and SEMG may be the possible technical solutions to this issue.

It is believed that with the continuous advancement of science and technology, the stimulation parameter research of MA should have more feasible solutions. The establishment of the stimulation parameter system can provide a more objective comparison criteria for the effectiveness evaluation of education, clinical treatment, and experimental intervention of MA.

## Figures and Tables

**Table 1:** Stimulation parameters can be the reference for the parameter system.

Parameter type	Parameters
Manipulation parameter	Selection of different acupuncture manipulations^†^
Kinematics parameter	Amplitude, velocity, and acceleration along *X*, *Y*, and *Z* axes
	Torsion angle, angular velocity, and angular acceleration
Kinetics parameter	Components along *X*, *Y*, and *Z* axes of finger force or tissue resistance
	Centripetal force during twirling method
	Power generated by finger force or tissue resistance
Time parameter	Treatment cycle and frequency^‡^
	Manipulation cycle and frequency
	Time course of the thrusting/lifting or left twirling/right twirling movements

*Note*. The meaning of some parameters is illustrated in Figure 3. ^†^Selection of acupuncture manipulations based on the patient's diagnosis including “lifting-thrusting” or “twirling” and “reinforcing” or “attenuating”. ^‡^Treatment cycle: a period of acupuncture treatment followed by a period of rest (no treatment) that is repeated on a regular schedule. Treatment frequency: the acupuncture treatment times in a week or month.

**Table 2:** The comprehensive comparison of different measurement technologies.

Measurement technologies	Measurable parameters		Advantages	Disadvantages
	Kinematic parameters	Kinetic parameters		
Sensors attached on needle (needle body modification)	Yes (motion sensor)	Yes (mechanical sensor)	(1) Real-time parameter acquisition	(1) Increasing the weight and volume of the needle
			(2) Measurable kinematics and kinetic parameters	(2) Affect the finger sensation.
Sensors surrounds needle (operating environment modification)	Yes	No	(1) Real-time parameter acquisition	(1) Performed on machine instead of human body
			(2) Maintain the naturalness of finger sensation	(2) No kinetic parameters
Motion-tracking technology	Yes	No	(1) Real-time acquisition of more parameters	(1) No kinetic parameters
			(2) Maintain the naturalness of tester's movement	(2) Easy to generate measurement errors
			(3) Performed on human body	
Virtual technology	Yes	No	(1) Full digitalization and real-time parameter acquisition	The virtual sensation is quite different from the actual operation
			(2) Realistic haptic feedback	
